# Sevelamer Attenuates Bioprosthetic Heart Valve Calcification

**DOI:** 10.3389/fcvm.2021.740038

**Published:** 2021-09-29

**Authors:** Zhen Meng, Zhe Li, Erli Zhang, Li Zhang, Qingrong Liu, Yongjian Wu

**Affiliations:** ^1^State Key Laboratory of Cardiovascular Disease, Fuwai Hospital, National Center for Cardiovascular Diseases, Chinese Academy of Medical Sciences and Peking Union Medical College, Beijing, China; ^2^Analytical Instrumentation Center, College of Chemistry and Molecular Engineering, Peking University, Beijing, China

**Keywords:** valvular heart disease, bioprosthetic heart valve, structural valve degeneration, sevelamer, anti-calcification

## Abstract

**Objective:** Sevelamer hydrochloride is a phosphate binder used to treat hyperphosphatemia in chronic kidney disease (CKD) patients that can reduce valvular and vascular calcification. The aim of this study was to examine the effects of sevelamer treatment on calcification in bioprosthetic heart valves (BHVs).

**Methods:** Wister rats were randomly divided into three groups according to sevelamer intake and implantation (sham–sham operation; implant–implantation and normal diet, implant+S implantation, and sevelamer diet). Two kinds of BHVs—bovine pericardium treated with glutaraldehyde (GLUT) or non-GLUT techniques—were implanted in rat dorsal subcutis at 4 weeks. After implantation, sevelamer was administered to the implant+S group. The animals were executed at days 0 (immediately after implantation), 7, 14, 28, and 56. Calcium levels were determined by atomic absorption spectroscopy and von Kossa staining. Serum biochemistry analysis, Western blotting, real-time quantitative polymerase chain reaction, alkaline phosphatase activity measurement, histopathologic analysis, immunohistochemistry, and enzyme-linked immunosorbent assay were conducted to identify the anti-calcification mechanism of sevelamer.

**Results:** Non-GLUT crosslinking attenuates BHV calcification. Serum phosphate and calcium remained unreactive to sevelamer after a 14-day treatment. However, the mean calcium level in the implant+S group was significantly decreased after 56 days. In addition, the PTH level, inflammatory cell infiltration, system and local inflammation, and expression of Bmp2, Runx2, Alp, IL-1β, IL-6, and TNF-α were significantly reduced in the implant+S group.

**Conclusion:** Sevelamer treatment significantly attenuated the calcification of BHVs and had anti-inflammation effects that were independent from serum calcium and phosphate regulation. Thus, sevelamer treatment might be helpful to improve the longevity of BHVs.

## Introduction

Valvular heart disease (VHD) is a common disease accounting for substantial morbidity and mortality in developed countries. According to a study in 2006, the prevalence of VHD is 2.5% in the US population (1). So far, no drug treatment can improve the long-term outcome of VHD compared with its natural history, and valve replacement surgery is the first-line therapy for the patients (2). Approximately 2,80,000 valvular substitutes are now implanted worldwide each year, with a predicted increment to 8,50,000 per year by 2050 (3). Currently available prosthetic valves can be divided into mechanical heart valves (MHVs) and bioprosthetic heart valves (BHVs). Each of the valves has inherent limitations. MHVs are durable yet highly thrombogenic, which requires lifelong anticoagulation therapy. BHVs do not require anticoagulant therapy but their durability is limited due to the inevitable structural valve degeneration (SVD) (2).

SVD, including calcification, leaflet fibrosis, tear, or flail leading to degeneration and/or hemodynamic dysfunction, is an intrinsic permanent change of BHVs. The rate of SVD is 10–30% at year 10 and 20–50% at year 15. Approximately 74% of valve failure requiring reoperation arises from SVD (4). As of today, there is no Food and Drug Administration-approved therapy to control SVD. SVD is believed to depend on the mechanical properties of the valve and the immunologic and calcification process (3). The general mechanisms of the native valve and BHV calcification seem to be related. Calcium and phosphate in the blood can form crystal cores in BHVs, which play important roles in BHV calcification (5). These findings indicated that attenuating the calcification might prolong the durability of BHVs (3).

Sevelamer hydrochloride (Renagel), a crosslinked poly(allylamine hydrochloride), hereafter referred to as sevelamer, is a non-absorbed calcium-free and aluminum-free phosphate binder used for the treatment of hyperphosphatemia in end-stage renal disease (ESRD) patients. A previous study has shown that sevelamer can reduce the calcification of vasculature and naïve aortic valves in chronic kidney disease (CKD) animal models and patients (6, 7). In addition, observational studies reported that a 0.1 mmol/L increase in serum phosphate was associated with a 50% increase in the risk of naïve aortic valve calcification (8). Based on these findings, we hypothesized that sevelamer could attenuate BHV calcification by decreasing serum phosphate levels in patients with normal renal function. In this study, we used a subdermal implantation model of normal rats to evaluate the effects of sevelamer therapy on post-implantation tissue changes in commercially available BHVs.

## Materials and Methods

### Materials

Commercial sevelamer hydrochloride (Renagel), a crosslinked poly(allylamine hydrochloride), was purchased from Sanofi. In addition, two kinds of commercial BHVs were kindly provided by the Peijia Medical Limited laboratory. Briefly, bovine pericardium (BP) sheets were treated with two different chemical treatment techniques as previously described (9): (a) BP sheets treated by glutaraldehyde (GLUT), and (b) BP sheets treated by an alternative, irreversible carbodiimide-based crosslinking chemistry (non-GLUT) (9). The rat diet was provided by Beijing HFK bioscience CO., LTD. containing 18% protein, 4% fat, 1.0–1.8% calcium, and 0.6–1.2% phosphorus. Sevelamer was administered together with the diet as a 3% w/w mix with animal chow as previously described (10).

### Experimental Design

This animal study was performed according to the guidelines for animal care and approved by the Animal Care and Utilization Committee, Experimental Animal Center, Fuwai Hospital, National Center for Cardiovascular Diseases, China. Ninety healthy specific-pathogen-free (SPF) 4-week-old male Wister rats weighing 50 ± 2.51 g purchased from Beijing Vital River Laboratory Animal Technology Corporation were used in the present study. The rats were housed in a specific-pathogen-free animal facility at 25°C with a 12-h light/dark cycle. The rats had free access to chow diets and water. These rats were randomly assigned to three groups with 30 animals in each: sham group (standard diet), implant group (standard diet), and implant+S group (standard diet with 3% w/w sevelamer). One GLUT cusp and one non-GLUT BHV cusp were surgically implanted in the dorsal subcutis of rats of implant group and implant+S group. In each group, five to six rats were euthanized by tribromoethanol, and samples were harvested at the designated time points (days 0, 7, 14, 28, and 56).

### Animal Operation

Four-week-old rats were euthanized by tribromoethanol (Sigma-Aldrich). One dorsal midline surgical incision was made, and two subdermal pockets were created on either side of the sagittal plane. For each rat of implant and implant+S groups, one GLUT and one non-GLUT BHV cusps (1 × 1 cm) were implanted and positioned to lie as flat as possible in each of the pockets. One sample was placed per pocket. The incision was closed with a surgical suture. For rats from the sham group, surgery was performed without implanting cusps. After surgery, 20,000 IU benzylpenicillin sodium was injected intramuscularly for injection prophylaxis for 3 days. After the designated period, the rats were euthanized by tribromoethanol. The whole blood was collected *via* post-cava. After clotting at room temperature, serum samples were obtained by centrifugation at 4,000 rpm for 30 min at 4°C and stored at −80°C until further analysis. The heart with the aortic root, kidneys, and thoracic aorta was harvested and fixed in 10% neutralized buffered formalin (Leagene Biotech, Beijing, China) for histological analysis. The BHV cusps and surrounding capsules were removed from the subdermal sites. The middle section was fixed in 10% neutralized buffered formalin for histological analysis. The remaining sections were placed immediately in liquid nitrogen and stored at −80°C as soon as possible for molecular analysis and quantitative calcium determination.

### Serum Biochemistry

Serum phosphate, calcium, alkaline phosphatase (ALP), and creatinine were measured by the Center of Laboratory Medicine, Fuwai Hospital. Briefly, phosphate was determined with a phosphate test kit (molybdate method) (Biosino, Beijing, China). Calcium was determined with a calcium test kit (o-Cresolphthalein complexone, OCPC method; Biosino, Beijing, China). Phosphate and calcium were measured using an AU 5800 automatic analyzer (Beckman Coulter, USA). ALP was determined with the ALP kit (NPP substrate-AMP buffer method; Biosino, Beijing, China). Creatine was determined with creatinine kit (Jaffé method; Biosino, Beijing, China). ALP and creatinine were measured using a Hitachi LABOSPECT 008AS automatic analyzer (Hitachi, Tokyo, Japan).

### Enzyme-Linked Immunosorbent Assay (ELISA)

Serum parathyroid hormone (PTH) interleukin-1β (IL-1β), interleukin-6 (IL-6), and tumor necrosis factor-α (TNF-α) were detected by ELISA (R&D Systems, MN, USA for IL-1β, IL-6, TNF-α; Meimian, Wuhan, China for PTH), according to the manufacturer's instructions. Dilutions and determination of standards were performed according to the manufacturer's instructions. The amounts of inflammatory markers in each serum sample were determined by matching the optical density in solution at 450 nm with a standard curve.

### Quantitative Calcium Determination

Quantitative calcium determination was performed by the Analytical Instrumentation Center, Peking University. Briefly, one section of each cusp (without surrounding connective tissue) was weighed and acid hydrolyzed in 6N Ultrex II HCl, dried under nitrogen gas, and resuspended in 6N Ultrex II HCl. Samples were centrifuged to remove the remaining particles and diluted in nano-filtered water. Calcium was analyzed using a prodigy 7 inductively coupled plasma-atomic emission (ICP-AES) spectrometer (Leeman, USA). Standards of calcium ions were prepared by diluting the commercial −1000 ppm ICP standards. The calibration was linear, with a maximum error of 5%. The solutions were diluted to appropriate concentration using 1% HNO_3_ (w/w). The intensity was obtained using a 10-s exposure, and the result was an average of three reads. Dilution ratios were used to calculate the calcium content of the sample, and values were normalized to dry sample weight.

### Histopathologic Analysis

Histopathologic analysis was performed by the Pathology Department of Fuwai Hospital. Rat heart, kidney, aorta, and BHV cusps fixed in 10% neutralized buffered formalin were embedded in paraffin. Sections of 5-μm thickness were stained with hematoxylin and eosin (Leagene Tech, Beijing, China). Histopathologic evaluation was performed by two experienced pathologists. The evaluation criteria are described in the Supplementary Material.

### Immunohistochemistry (IHC)

For IHC, endogenous peroxidase activity of paraffin-embedded sections was blocked with 0.3% hydrogen peroxide in methanol. Sections, each separated by 3–5 μm, were washed with PBS and incubated with rabbit anti-CD68 monoclonal antibody (Abcam, 1:500) to stain macrophages and rabbit anti-CD11b monoclonal antibody (Abcam, 1:500) to stain neutrophils at 4°C overnight. Specimens were then treated with a peroxidase conjugated goat anti-mouse secondary antibody (ZSGB Biotech, CA) and diaminobenzene substrate (ZSGB Biotech). Images were taken using a Leica DM 6000B microscope (Leica, Germany). The semi-quantitative analysis was performed by ImageJ analysis software.

### Von Kossa Staining

Paraffined sections were washed with distilled water and incubated with 5% aqueous AgNO_3_ (Abcam) under ultraviolet light for 45 min, then treated with 2.5% sodium thiosulfate (Abcam) for 2 min, and finally incubated with 0.1% nuclear fast red (Abcam) for 1 min. Images were taken using a panoramic SCAN image system (3DHISTECH). The semi-quantitative analysis was performed by ImageJ analysis software.

### Real-Time Quantitative Polymerase Chain Reaction (RT-qPCR)

Total RNAs were isolated from BHV cusps with surrounding capsules using a RNeasy mini kit (Qiagen) following the manufacturer's instructions. Reverse transcription (RT) was performed using PrimeScript™ RT Master Mix (Takara, CA, USA). qPCR was performed with SYBR™ Green Master Mix (Applied Biosystems) and run on 7500 or QuantStudio 3/5 Real-Time PCR systems (Applied Biosystems). Rat Gapdh was used as internal control unless specified otherwise. RT-qPCR assay primers used in this study are listed in Table 1.

**Table 1:** qPCR primers used in this study.

**Gene**	**Forward**	**Reverse**
BMP2	TCTGGAAGCTGTGGGATAGA	GAGGAGCCTGTGGAGAAATAC
RUNX2	GACTGTGGTTACCGTCATGGC	ACTTGGTTTTTCATAACAGCGGA
IL-1β	ATGGCAACTGTCCCTGAACTCAACT	CAGGACAGGTATAGATTCAACCCCTT
TNF-α	GCAGATGGGCTGTACCTTATC	GAAATGGCAAATCGGCTGAC
IL-6	GTTGCCTTCTTGGGACTG	ACTGGTCTGTTGTGGGTG
GAPDH	GGAGTCTACTGGCGTCTTCAC	ATGAGCCCTTCCACGATGC

*Runx2, RUNX family transcription factor2; Bmp2, bone morphogenetic protein 2, IL-1β, interleukin 1 beta; IL-6, interleukin 6; TNF-α, tumor necrosis factor-alpha; Gapdh, glyceraldehyde-3-phosphate dehydrogenase*.

### Western Blot

The proteins of BHV cusps and the surrounding capsules were extracted with RIPA Lysis Buffer (Beyotime). The proteins were boiled with SDS-PAGE sample loading buffer for 5 min. The samples were then loaded onto a NuPAGE Bis-Tris gel (Thermo) followed by electrophoresis and transferred onto PVDF membranes. Membranes were blocked with TBST containing 5% milk powder, incubated with rabbit anti-RUNX2, BMP2, and GAPDH (Abcam) antibodies overnight at 4°C. The membranes were then washed and incubated with HRP-labeled goat anti-rabbit secondary antibodies (Beyotime Biotech). Detection was done using SuperSignal West Femto Maximum Sensitivity Substrate (Thermo Fisher Scientific). The images were acquired using the FluorChem M system (ProteinSimple). Quantification analyses were performed using ImageJ analysis software.

### ALP Activity Measurement

The proteins of BHV cusps and the surrounding capsule were extracted with RIPA Lysis Buffer (Beyotime). The ALP activity was measured with Alkaline Phosphatase Assay Kit (Beyotime) following the manufacturer's instructions. Briefly, certain dilutions of samples were incubated with para-nitrophenyl phosphate to produce p-nitrophenol in diethanolamine (DEA, pH 9.8) buffer. The amount of ALP activity in each serum sample was determined by matching the optical density in the solution at 405 nm with a standard curve. A series dilution of p-nitrophenol (10 mM) were used to create a standard curve.

### Statistics

Continuous data were expressed as mean ± standard deviation (SD) and compared using Student's *t*-test (when two groups were compared) or one-way analysis of variance with Dunnett's or Tukey's multiple comparisons tests (when more than two groups were compared). Normal data distribution was checked using the Shapiro–Wilk test. Finally, categorical data were expressed as sample numbers and compared with Kruskal–Wallis one-way analysis of variance. A *p*-value < 0.05 for the two-sided test was considered statistically significant. All analyses were performed with IBM SPSS statistics 25 and GraphPad Prism 8.0.

## Results

### Effect of Sevelamer on Serum Biochemistry and Body Weight

No difference was observed between the three groups in food intake and body weight (data not shown). No abnormality was observed in the heart, kidneys, and thoracic aorta of rats treated with sevelamer. No difference was observed between sham and implant groups in the concentrations of serum phosphate, calcium, calcium-phosphate product (Ca × P), ALP, PTH, and creatine, except for a transient increment of ALP at day 7 because of stress response to the implantation (Supplementary Figure 1).

**Figure 1:**
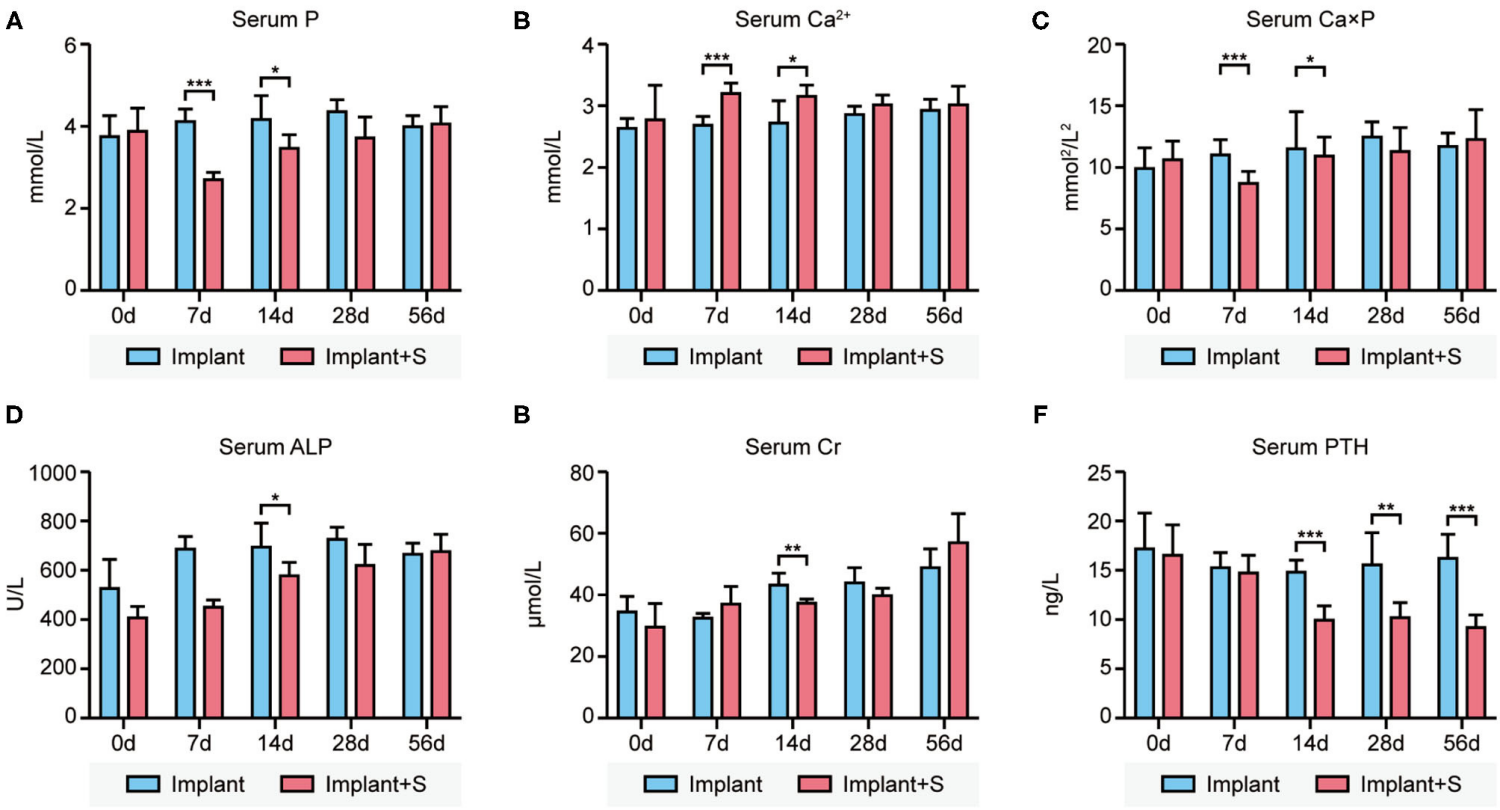
Difference of serum biochemistry parameters between the implant and implant+S groups at days 0, 7, 14, 28, and 56 after implantation. **(A)** Compared to implant and implant+S group concerning serum phosphate levels, serum phosphate level decreased transiently in implant+S groups in 7–14 days. **(B)** Comparison between the implant and implant+S group concerning serum calcium levels. Serum calcium level increases transiently in the implant+S group in 7–14 days. **(C)** Comparison between the implant and implant+S group concerning serum calcium-phosphate product; serum calcium-phosphate product level decreased transiently in the implant+S group at 7 days. **(D)** Comparison between the implant and implant+S group concerning serum ALP. **(E)** Comparison between the implant and implant+S group concerning serum creatine. **(F)** Comparison between the implant and implant+S group concerning serum PTH level; serum PTH was decreased continuously in the implant+S group after 14 days. *N* = 4–6, data are mean ± SD, *p < 0.05, **p < 0.01, ***p < 0.001.

Serum phosphate was significantly lower in the implant+S group than in the implant group at days 7 and 14. In contrast, serum calcium was significantly higher in the implant+S group than the implant group at days 7 and 14. Ca × P was decreased in the implant+S group at 7 days. At day 14, serum ALP and creatine was slightly lower in the implant+S group than in the implant group. All differences mentioned above disappeared after 28 days (Figures 1A–E). However, serum PTH decreased continuously in the implant+S group than in the implant group after 14 days (Figure 1F).

### Effect of Sevelamer on Calcium Levels in the Implants

**Figure 2:**
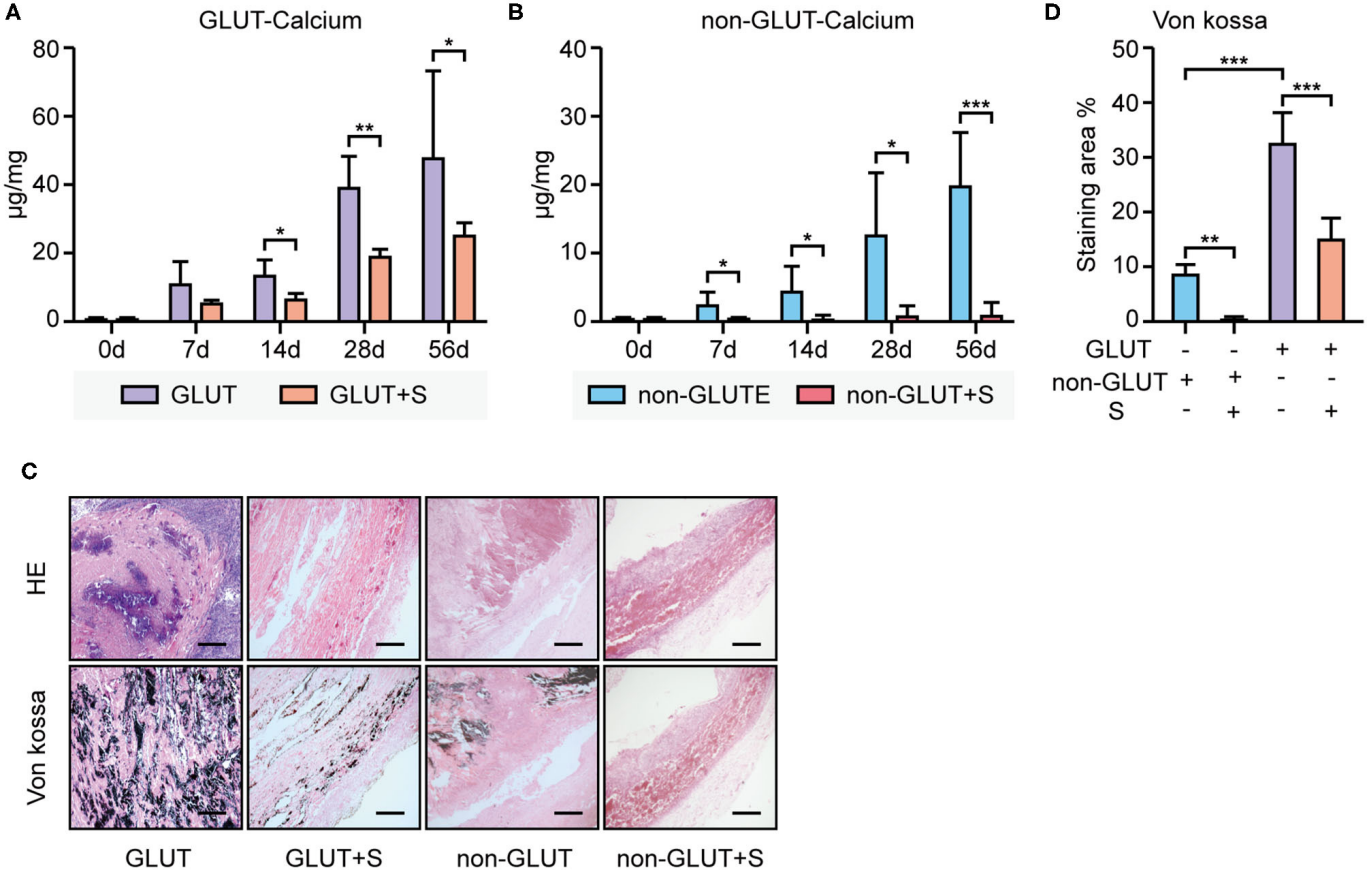
Calcium deposition analysis of BHVs between the implant and implant+S group. **(A)** Calcium levels of GLUT BHVs between the implant and implant+S group, at days 0, 7, 14, 28, and 56 after implantation. **(B)** Calcium level of non-GLUT BHVs between the implant and implant+S groups at days 0, 7, 14, 28, and 56 after implantation. **(C)** Representative images and quantification of von Kossa staining and hematoxylin–eosin (HE) staining of BHVs in implant and implant+S groups 56 days after implantation; scale bars represent 400 μm. **(D)** Quantification of von Kossa staining of BHVs in implant and implant+S groups 56 days after implantation. GLUT represents GLUT BHVs in the implant group, GLUT+S represents GLUT BHVs in the implant+S group, non-GLUT represents non-GLUT BHVs in the implant group, and non-GLUT+S represents non-GLUT BHVs in the implant+S group. *N* = 4–6, data are mean ± SD, *p < 0.05, **p < 0.01, ***p < 0.001.

The calcium level was quantified by ICP-AES. The calcium level of GLUT BHVs was significantly higher than that of non-GLUT BHVs in the implant group after 56 days (GLUT vs. non-GLUT calcium: 47.44 ± 25.49 vs. 19.89 ± 7.95 μg/mg), suggesting that non-GLUT linking BHV implantation resulted in fewer valvular calcification. Accordingly, we evaluated the effect of sevelamer in rats implanted with GLUT and non-GLUT linking BHVs. Furthermore, BHVs of the implant group had a higher calcium level than that of the implant+S group (Figures 2A,B). The implant+S group showed significantly lower levels of *de novo* BHV calcification at all measured time points from 7 to 56 days. Heavy calcification on BHVs in the implant group was verified by von Kossa staining 56 days after implantation (Figure 2C). von Kossa staining of BHVs in the implant+S group showed less calcification after implantation (Figure 2C). These results were confirmed using a quantitative analysis (Figure 2D).

### Effect of Sevelamer on the Expression of Osteoblast Markers in the Implants

**Figure 3:**
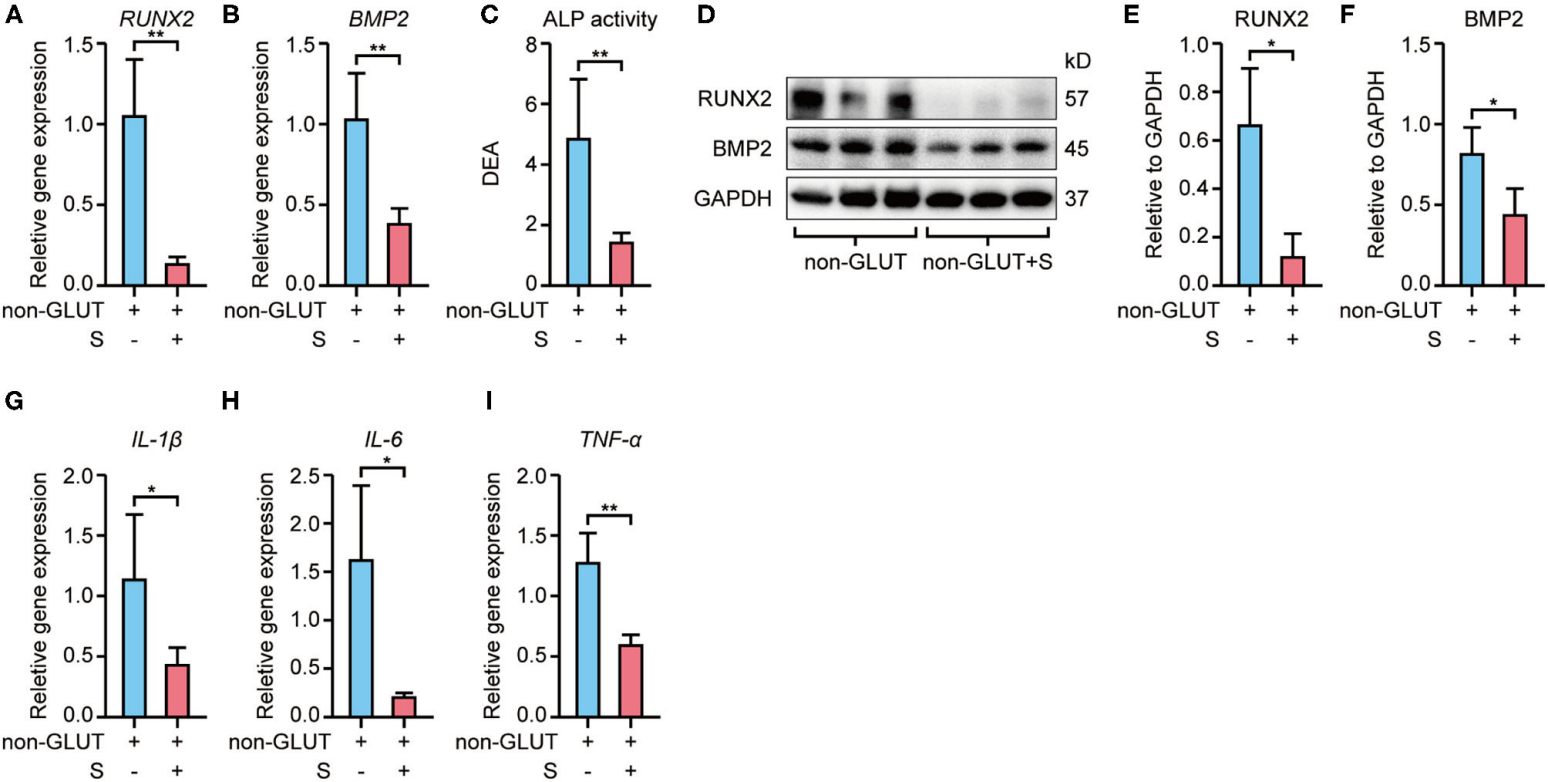
Expression of osteoblast and inflammatory markers of non-GLUT BHVs in implant and implant+S groups 56 days after implantation. **(A,B)** Sevelamer reduces Runx2 and Bmp2 mRNA levels. **(C)** Sevelamer reduces ALP activity. **(D–F)** Protein expression of RUNX2 and BMP2 quantification analysis **(E,F)** and representative blotting **(D)**. **(G–I)** mRNA expression of IL-1β **(G)**, IL-6 **(H)**, and TNF-α **(I)** decreased in the implant+S group. *N* = 3 for Western blot, *N* = 4 for RT-qPCR, *N* = 5 for ALP activity. non-GLUT represents non-GLUT BHVs in the implant group, and non-GLUT+S represents non-GLUT BHVs in the implant+S group. Data are mean ± SD, *p < 0.05, **p < 0.01.

**Figure 4:**
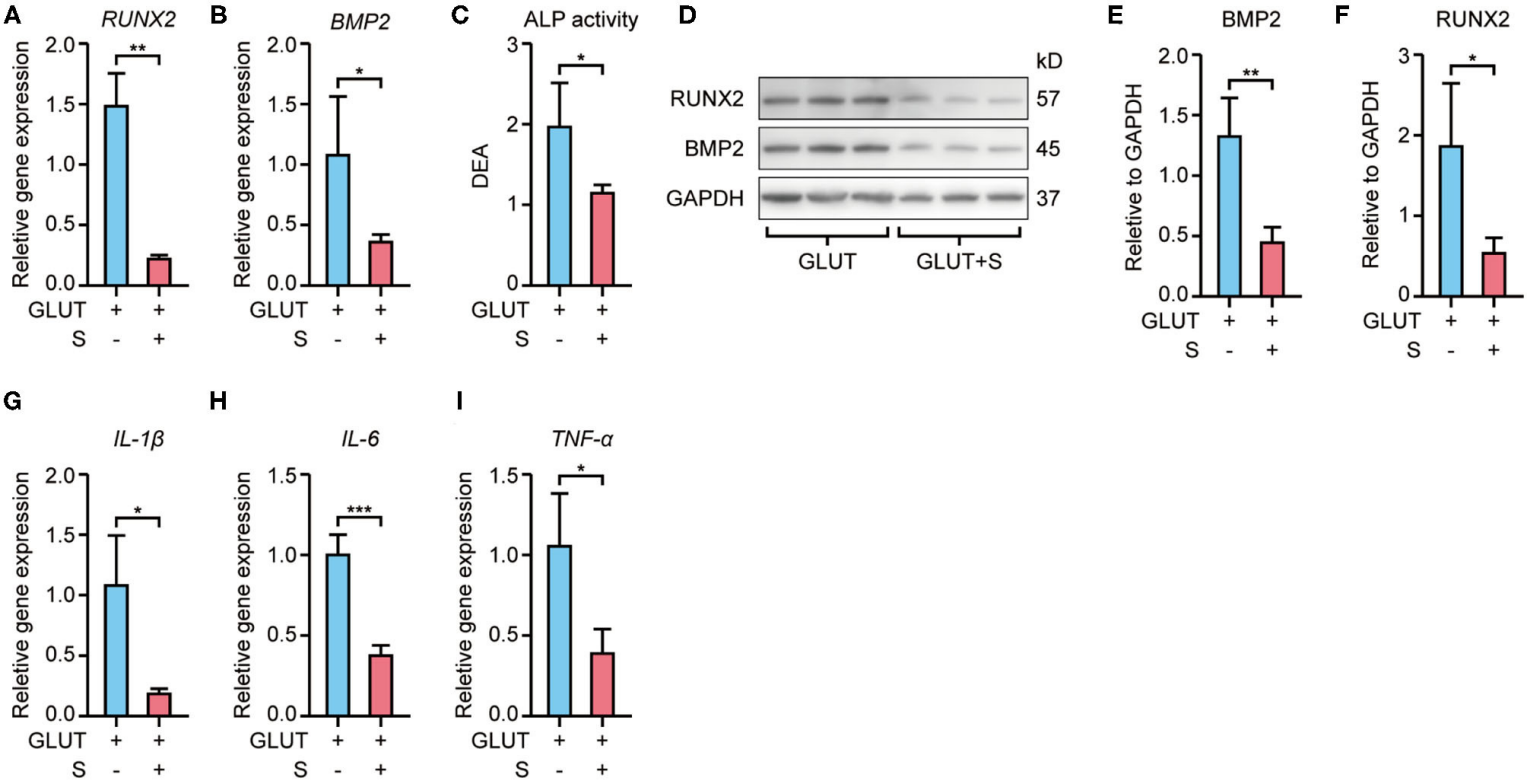
Expression of osteoblast and inflammatory markers of GLUT BHVs in implant and implant+S groups 56 days after implantation. **(A,B)** Sevelamer reduces Runx2 and Bmp2 mRNA levels. **(C)** Sevelamer reduces ALP activity. **(D–F)** Protein expression of RUNX2 and BMP2 quantification analysis **(E,F)** and representative blotting **(D)**. **(G–I)** mRNA expression of IL-1β **(G)**, IL-6 **(H)**, and TNF-α **(I)** decreased in the implant+S group. *N* = 3 for Western blot, *N* = 4 for RT-qPCR and ALP activity. GLUT represents GLUT BHVs in the implant group, and GLUT+S represents GLUT BHVs in the implant+S group. Data are mean ± SD, *p < 0.05, **p < 0.01, ***p < 0.001.

RT-qPCR revealed that Runx2 and Bmp2 mRNA expression were higher in BHVs of the implant group than the implant+S group on the 56th day after implantation (Figures 3A,B, 4A,B). Western blot confirmed these changes in protein level (Figures 3D–F, 4D–F). In addition, the ALP activity measure showed that BHVs of the implant group have a higher ALP activity than those of the implant+S group (Figures 3C, 4C) on the 56th day after implantation.

### Effect of Sevelamer on the Local and Systematic Inflammatory

**Figure 5:**
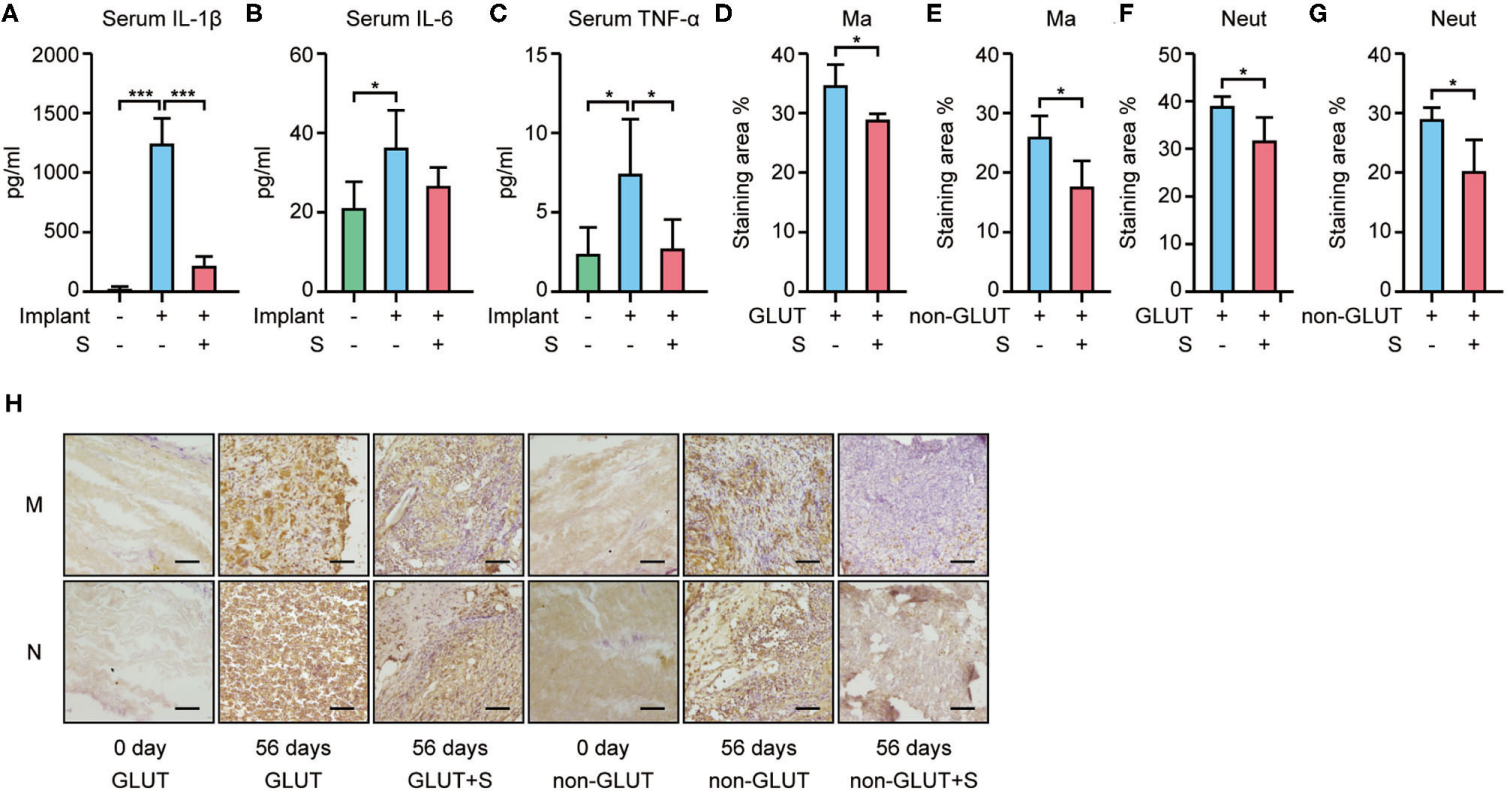
Inflammatory markers in serum and macrophage/neutrophil infiltration in BHVs in implant and implant+S groups 56 days after implantation. **(A–C)** Serum level of IL-1β **(A)**, IL-6 **(B)**, and TNF-α **(C)** increased in the implant group and attenuated by sevelamer. **(D–H)** Macrophage and neutrophil infiltration in BHVs and attenuated by sevelamer, and representative images **(H)** and quantification analysis of IHC staining; scale bars represent 100 μm **(D–G)**. GLUT represents GLUT BHVs in the implant group, GLUT+S represents GLUT BHVs in the implant+S group, non-GLUT represents non-GLUT BHVs in the implant group, and non-GLUT+S represents non-GLUT BHVs in the implant+S group. M/Ma represents macrophage, and N/Neut represents neutrophil. *N* = 4, data are mean ± SD, *p < 0.05, ***p < 0.001.

RT-qPCR showed that BHVs of the implant group have higher levels of the inflammatory marker, including IL-1β, IL-6, and TNF-α, than those of the implant+S group on the 56th day after implantation (Figures 3G–I, 4G–I). After 56 days of implantation, IL-1β, IL-6, and TNF-α in serum were significantly increased in the implant group than the sham group (Figures 5A–C). However, sevelamer significantly suppressed the increments of IL-1β, IL-6, and TNF-α in serum (Figures 5A–C). Pathological analysis and immunohistochemistry showed that BHVs of the implant group have higher levels in degeneration, granulation tissue invasion, neutrophils, and macrophages than that of the implant+S group at day 56 after implantation (Table 2; Figures 2C, 5D–H).

**Table 2:** Histopathologic analysis of non-GLUT BHVs in implant and implant+S groups after 56 days of implantation.

	**Class**	**Implant+S**	**Implant**	***p*-value**
Number		5	5	
Degeneration	-	5	0	0.003
	I	0	0	
	II	0	0	
	III	0	0	
	IV	0	5	
Necrosis	-	4	0	0.063
	I	0	0	
	II	0	1	
	III	0	2	
	IV	1	2	
Calcification	-	3	0	0.163
	I	1	2	
	II	0	1	
	III	0	2	
	IV	1	0	
GranulationTissue invasion	-	0	4	0.011
	I	2	1	
	II	3	0	
	III	0	0	
	IV	0	0	
Neutrophil	-	4	0	0.007
	I	1	0	
	II	0	2	
	III	0	1	
	IV	0	2	
Macrophage	-	2	0	0.033
	I	3	2	
	II	0	1	
	III	0	2	
	IV	0	0	

## Discussion

Studies have reported that sevelamer can lower serum phosphate concentration and thus attenuate the progression of cardiovascular calcification in both CKD animal models and real-world patients (11, 12). The present study demonstrated that 3% w/w sevelamer supplementation in chow diet was sufficient to reduce bioprosthesis calcification in subdermal BHV implantation rat models. However, sevelamer did not significantly affect the levels of serum phosphate, calcium, calcium-phosphate products, serum ALP activity, and creatinine in rats implanted with non-GLUT linking BHVs at day 56 of treatment, but caused transient fluctuations of these parameters between days 7 and 14. Our further research confirmed that this anti-calcification function may resort to decreasing PTH levels, attenuating local and system inflammatory and downregulating factors in the calcification signaling pathway, including BMP2, RUNX2, and ALP.

As sevelamer is not absorbed from the gut, it cannot induce hypophosphatemia and direct effects on the vascular system. Our study showed that sevelamer does not affect body weight and food intake, corroborating previous studies (13). Bone parameters were not measured in this study, but according to the literature, sevelamer cannot reduce bone density (5, 14). Since sevelamer cannot be absorbed in the gut and cannot induce hypophosphatemia and hypocalcemia, we can conclude that sevelamer is a safe treatment to attenuate BHV calcification.

Approximately 20% of cardiac surgery is used to treat VHD, and about half of the valve replacements use BHVs (15). BHVs are recommended strongly especially in patients with high bleeding risk and young women contemplating pregnancy (2). Up to now, three basic types of BHVs exist: xenografts, homografts, and autografts. Xenografts account for most of BHVs since biomaterials of animal origin are widely available. Most commercially available BHVs are fabricated out of porcine aortic valve leaflets or BP sheets crosslinked with glutaraldehyde (GLUT). GLUT crosslinking can make BHVs resistant against enzymatic degradation and reduce tissue antigenicity. However, GLUT-crosslinked BHVs experience failure due to calcification or degeneration within 10–20 years and seem to fail faster in younger patients (15). BHV calcification shares some general mechanisms with native aortic valve calcification involving passive and active mechanisms. The passive mechanism prosthesis-related dystrophic calcification is due to precipitation of calcium phosphate on cell debris and fibrous. The active mechanism means valvular infiltration of cell or cell elements (calcium-binding proteins, macrophage, T lymphocyte, and matrix metalloproteinase). Several studies have suggested an association between inflammation, reactive oxygen species, thrombogenicity, coagulation, and calcification. Mechanical stress and cyclic loading are more likely to cause BHV calcification than native valves because, structurally, BHVs are different from native valves. The immune response to xenograft antigens such as galactose-α-1,3-galactose (α-Gal) and N-glycolylneuraminic acid (NeuGc), which are not expressed in humans, contributes to BHV calcification. Clinical studies also documented the relationship between BHV calcification and impaired lipid metabolism (3, 5).

GLUT crosslinking does not stabilize components such as elastin and glycosaminoglycans (GAGs) of extracellular matrix (ECM), making BHVs rigid and more prone to mechanical calcification degeneration than normal valves. Calcification has been shown to originate at devitalized cells and damaged ECM components (4). We test a new generation of non-GLUT BHVs provided by Peijia Medical Ltd. These BHVs use an alternative, irreversible carbodiimide-based crosslinking chemistry to stabilize more of the ECM component such as elastin and GAGs and produce stronger and more compliant BHVs (9). In our study, non-GLUT BHVs are less prone to calcification than traditional GLUT BHVs in subdermal implantation animal models, demonstrating that non-GLUT BHVs may be more durable heart valve implants in future.

A previous study has reported that 1% w/w sevelamer did not show any effect on serum phosphate in normal rat and normal rats treated with 3% w/w sevelamer showed a significant decrease in serum phosphate in 2–8 days (11). Since many studies chose 3% w/w sevelamer to treat rats (6, 11, 13), we decided to follow these studies. However, dosages of sevelamer should be screened in further studies to find an optimal dosage. Several studies showed that sevelamer has no effect on serum phosphate and calcium level of normal rat models after weeks of treatments (6, 13). A clinical study also failed to detect a significant decrease in serum phosphate level in patients with aortic stenosis using sevelamer for 6 weeks (8). These studies are consistent with our finding that sevelamer can only cause a transient decrease of serum phosphate levels in normal rats. Serum phosphate decreased transiently after sevelamer treatment in 7–14 days. Meanwhile, serum calcium increased to maintain the calcium-phosphate product. However, these changes disappear after 14 days. A clinical study showed that phosphate excretion from urine reduced during sevelamer treatment periods in the non-CKD population (8). We speculated that serum calcium and phosphate returning to the normal level could be attributed to renal and endocrine regulation. Our study found that PTH levels in the implant+S group consistently decreased after 14 days. Calcium and phosphate in the blood can support the growth of calcium phosphates on cell debris and fibrous components of BHVs, although serum calcium and phosphate are unchanged during most periods of sevelamer treatment. Sevelamer's influence on phosphate metabolism may affect the passive mineralization of BHVs, which needs further studies.

A relationship between inflammation and calcification has been demonstrated in many studies (3, 5). Macrophage is the most found immune cell in BHVs, whereas T lymphocytes and B lymphocytes, neutrophils, and eosinophils are less frequent. The macrophage can produce matrix metalloproteinases (MMPs), reactive oxygen species, multiple cytokines, and chemokines to promote calcification. Besides local inflammation, system inflammation also plays an important role in BHV calcification. C/T genotype of the rs 2229238 polymorphism within the IL6R gene has been proved to be associated with an increased risk of severe BHV calcification, as the genotype was significantly associated with a higher IL-6 level (16). Previous studies have shown that sevelamer can decrease serum levels of C-reactive proteins in CKD patients (17, 18). Sevelamer-treated uremic rats have less interstitial fibrosis and inflammation (13). Our study found that sevelamer can reduce macrophage and neutrophil infiltration in BHVs and inflammatory cytokine levels, including IL-1β, IL-6, and TNF-α. Serum IL-1β in both BHVs and serum suggested that sevelamer can attenuate local and system inflammation. The anti-inflammation effect may explain the reason for the anti-calcification effect of sevelamer. In addition, a marked reduction of calcium deposition and lower expressions of osteoblast markers such as BMP2, RUNX2, and ALP in mRNA and protein levels were found in the sevelamer-treated group.

The relationship between BHV calcification and impaired lipid metabolism has also been documented. A clinical study showed that increasing serum cholesterol levels are associated with increased BHV calcification. The odds ratio for valve explanation was 3.9-fold higher in patients with serum cholesterol levels higher than 200 mg/dl (19). Animal studies showed that statin significantly attenuated calcification of bovine pericardial valves in a subcutaneous rat model (20). However, no clinical data supported the anti-calcification effects of statin in BHVs. Dysfunctional BHVs display a considerable lipid deposition and foam cells. Oxidized LDL (low-density lipoprotein) can be produced by reactive oxygen species released by infiltrating neutrophils and macrophages (3). Studies have confirmed that sevelamer can reduce total and low-density lipoprotein cholesterol (LDL-C) levels. Wilkes et al. reported a 35.9% decrease in serum LDL-C in hemodialysis patients treated with sevelamer (21). Sevelamer can also attenuate oxidative stress (22). However, our subdermal implantation model used a widely used rat without a gene modification strain, with a normal diet. Thus, it is a limited model to study the effect of sevelamer on BHV calcification resorting of regulation lipid metabolism. Sevelamer also has pleiotropic effects such as decreasing the phosphaturic hormone fibroblast growth 23 (FGF23), which is secreted by osteocytes and osteoblasts in response to oral phosphate loading and is associated with the development of endothelial dysfunction and cardiac hypertrophy in CKD (23) and regulation of the expression of cell cycle proteins (6), among other effects. More studies are required to explore the mechanism of the anti-calcification effect of sevelamer in BHVs.

Several techniques are being developed to avoid BHV calcifications (5, 15, 24), including a new crosslinking method to stabilize more ECM components, a decellularization technique to eliminate cellular debris, proper heart valve tissue sources from gene knockout animals with no immunological antigens, such as α-Gal and NeuGc, and new implantation techniques. However, considering that millions of BHVs have already been implanted, a therapy for BHV calcification prevention is required. Currently, there is no FDA-approved treatment to manage BHV calcification. Apart from a statin, anticoagulant therapy, immunosuppressive therapy, and MMP inhibitors under exploration to prevent BHV calcification (3), our study provided a new choice to prevent BHV calcification. However, more studies are needed to prove the effect.

## Limitation and Conclusion

This study has several limitations. First, we used a subcutaneous animal model that is different from the blood circulation model. However, this is the traditional first step when developing tissue-engineered heart valves in the rat, which is an animal model that is considerably easier and less expensive than the larger animal models. Second, we only used two types of BHVs and one dosage of sevelamer. More commercial BHVs and dosages of sevelamer need to be tested in the future. Third, sevelamer has many effects besides the anti-inflammatory action. More studies are needed to identify these effects on the prevention of BHV calcification. Finally, the relationship between the animal model and clinical outcomes has not been established. More clinical studies, including randomized controlled trials, are needed to prove the clinical value of sevelamer in the prevention of BHV calcification.

In conclusion, sevelamer treatment significantly attenuated the calcification of BHVs in a subcutaneous rat model and had anti-inflammation effects, which are independent from serum calcium and phosphate regulation. Thus, our results suggest that sevelamer treatment might be helpful for the prevention of BHV calcification.

## Data Availability Statement

The original contributions presented in the study are included in the article/Supplementary Material, further inquiries can be directed to the corresponding author.

## Ethics Statement

The animal study was reviewed and approved by the animal care and utilization committee, experimental animal center, Fuwai hospital National Center for Cardiovascular Diseases, China.

## Author Contributions

ZL and EZ contributed to the design and manuscript preparation of this study. ZM and QL contributed to the animal experiment, molecular study, and data collection. LZ contributed to the ICP-AES experiment. YW is an expert in valvular heart disease field who gave knowledge support to the study. All authors contributed to the article and approved the submitted version.

## Funding

This work was supported by grants from the National Natural Science Foundation of China (NSFC) (81700405) and China Scholarship Committee (CSC) award 201406210335 to EZ, from NSFC (91339103) to YW, Capital health development scientific research program (W01-ZD-02_W_2019) to YW, and from Special Research Fund for Central Universities, Peking Union Medical College (3332018052) to ZL.

## Conflict of Interest

The authors declare that the research was conducted in the absence of any commercial or financial relationships that could be construed as a potential conflict of interest.

## Publisher's Note

All claims expressed in this article are solely those of the authors and do not necessarily represent those of their affiliated organizations, or those of the publisher, the editors and the reviewers. Any product that may be evaluated in this article, or claim that may be made by its manufacturer, is not guaranteed or endorsed by the publisher.

## Abbreviations

VHD: valvular heart disease
MHV: mechanical heart valve
BHV: bioprosthetic heart valve
SVD: structural valve degeneration
CKD: chronic kidney disease
ESRD: end-stage renal disease
BP: bovine pericardium
GLUT: glutaraldehyde
ALP: alkaline phosphatase
RUNX2: RUNX family transcription factor 2
BMP2: bone morphogenetic protein 2
IL-1β: interleukin 1 beta
IL-6: interleukin 6
TNF-α: tumor necrosis factor-alpha
PTH: parathyroid hormone
GAPDH: glyceraldehyde-3-phosphate dehydrogenase
LDL-C: low-density lipoprotein cholesterol.
