# Supplementary material for: Sevelamer Attenuates Bioprosthetic Heart Valve Calcification
Source: Front Cardiovasc Med. 2021 Sep 29;8:740038. doi: 10.3389/fcvm.2021.740038 (PMC8514018; doi:10.3389/fcvm.2021.740038)

Supplementary Material

1. **Supplementary material**

**Histopathologic analysis criterion**

Evaluation of thrombus refers to national standard: Appendix B (informative annex) of “Infusion, transfusion, injection equipment for medical use -- Part 2: Biological test methods” (GB/T 14233.2-2005) (for more details: http://www.gb688.cn/bzgk/gb/newGbInfo?hcno=918417BDDA4BC3A49DBAE133B2F2E5FE).

Evaluation of local effects after implantation refers to national standard: “Biological evaluation of medical devices—Part 6: Tests for local effects after implantation” (GB/T16886.6—2015/ISO10993-6:2007). Local effects include fibrous capsule, degeneration, numbers, and distribution of inflammatory cells (neutrophil, lymphocyte, plasmacyte, eosinophils, and macrophage), necrosis, and neovascularization (for more details:http://www.gb688.cn/bzgk/gb/newGbInfo?hcno=BD6F4C0B3593497FF97782CFE88F7963).

Classification of calcification: class 0 – no calcification; class 1 – slight calcification, less than 25% of the material area; class 2 – medium calcification, 25–50% of the material area; class 3 – severe calcification, 50–75% of the material area; class 4 – very severe calcification, more than 75% of the material area.

Classification of granulation tissue invasion: class 0 – no granulation invasion; class 1 – focal granulation invasion does not reach 50% of material thickness; class 2 – focal granulation invasion reaches 50% of material thickness, or extensive granulation invasion does not reach 50% of material thickness. Class 3 – extensive granulation invasion reaches 50% of material thickness.

**2．Supplementary figure**

**Supplementary FIGURE S1 |** Difference of serum biochemistry parameters between sham and implant at days 0, 7, 14, 28, and 56 after implantation. **(A)**: Comparison between sham and implant groups concerning serum phosphate levels. **(B)**: Comparison between sham and implant groups concerning serum calcium levels. **(C):** Comparison between sham and implant groups concerning serum calcium-phosphate product. **(D)**: Comparing sham and implant groups with respect to serum ALP, serum ALP levels increase transiently in the implant group at seven days. **(E)**: Comparison between sham and implant groups with respect to serum creatine. **(F)**: Comparison between sham and implant group with respect to serum PTH level. N=4–6, data are mean±SD, *P<0.05, **P<0.01, ***P<0.001.


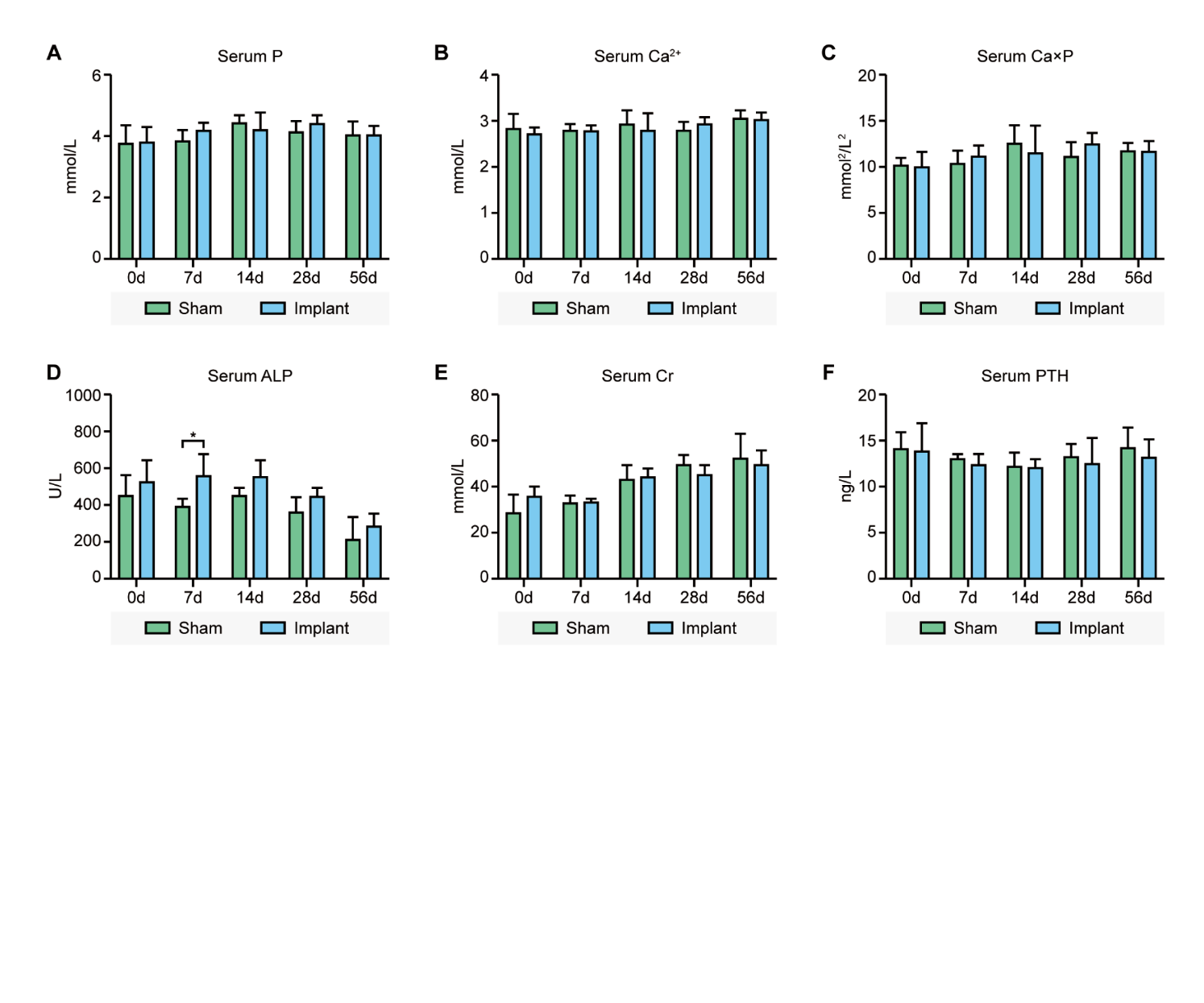

Supplement: Supplementary file 1 [file Data_Sheet_1.docx]
